# Combination therapy of phytonanomedicine and post-biotic for the management of Parkinson’s disease

**DOI:** 10.17179/excli2024-7770

**Published:** 2024-10-10

**Authors:** Bushra Bashir, Sukriti Vishwas, Monica Gulati, Gaurav Gupta, Sachin Kumar Singh

**Affiliations:** 1 School of Pharmaceutical Sciences, Lovely Professional University, Phagwara, Punjab, India; 2 Faculty of Health, Australian Research Center in Complementary and Integrative Medicine, University of Technology Sydney, Ultimo, NSW 2007, Australia; 3 Center of Medical and Bio-allied Health Sciences Research, Ajman University, Ajman, United Arab Emirates; 4 Center for Research Impact & Outcome, Chitkara College of Pharmacy, Chitkara University, Punjab, India

## ⁯⁯

Parkinson's Disease (PD) is a debilitating neurodegenerative condition that affects around 11 million individuals worldwide with 1 % of the population over 60 years old suffering from it with 1.5 times more preponderance of males over females (Je et al., 2021[5]). The rate of the infection ranges from 100 to 200 per 100,000 individuals and the yearly rate is assessed to be 15 per 100,000 individuals (Alves et al., 2009[1]; Brakedal et al., 2022[2]). It accounts to the second most prevalent neurological illness after Alzheimer's disease (AD), with median age-standardized annual incidence rates of 14 per 100,000 persons in the entire population in high-income nations, and 160 per 100,000 people aged 65 or older. Until presently the etiology of the illness remains elusive and is most likely accepted to be the cumulative effect of various environmental factors like toxins, heavy metals, sedentary lifestyle, pesticide exposure, well water, solvents, and hereditary inclinations involving α-synuclein, DJ-1, PINK1, Parkin, LRRK2, ATP13A2 and aging in people along with their interaction within the setting of brain aging (Wei et al., 2018[14]). However, the exact corroboration is sparse and inconclusive. A few pathways have been intimated in both heritable and sporadic shapes of PD such as incorporate mitochondrial failure, oxidative stress, motor circuit dysfunction, neuroinflammation, and α- synuclein homeostasis. 

Current medications only give symptomatic alleviation and do not prevent dopaminergic neuron loss. Individuals frequently see a noticeable improvement in their side effects after beginning levodopa/carbidopa treatment (Poewe et al., 2010[10]). They may, however, need to progressively raise the dose to get the best results. Although levodopa is typically so powerful that some people may not have symptoms in the early stages of the illness as long as they take the medication, it is not a cure. Although it can help with the symptoms of PD, it does not restore destroyed nerve cells or halt the disease from progressing. Moreover, the undesirable side effects, including cardiac arrhythmias, motor fluctuations, hallucinations, psychosis, constipation, blurred vision, nausea, vomiting, exacerbation of angina, anomalous developments (dyskinesias), behavioral impacts, and variance in motor performance are the major drawbacks of levodopa (Rus et al., 2022[11]). No remedy for PD exists nowadays, but research is progressing and medicines or surgery can regularly give considerable improvement in motor symptoms. Furthermore, metagenomic research has demonstrated over a decade the critical function of the gut microbiota in the upkeep of the immune and neuroendocrine systems and the employment of the post-biotic in the treatment of neurodegenerative diseases. Butyric acid as a post-biotic has shown neuroprotective activity by reducing neuroinflammation, and oxidative stress and decreasing the expression of α-synuclein in substantia nigra ameliorating motor and behavioral deficits.

Hops (Humulus lupulus L.), a reputed bittering agent applicable in the brewing industry, has been employed in traditional medicine. Xanthohumol (XH) is a plant based bioactive compound that has very good therapeutic potential for treating cancer, diabetes as well as neurodegenerative diseases such as Parkinson's and Alzheimer's disease. XH [3,3-dimethyl allyl]-2,4,4-trihydroxy-6 -methoxy chalcone) is the main prenylated flavonoid found in the female inflorescences of the hop plant ('hops'), which is used in the production of beer. Studies have reported various pharmacological activities of XH such as neuroprotective (Wang et al., 2019[13]), anticancer (Jiang et al., 2018[6]), antioxidant (Hartkorn et al., 2009[4]), anti-inflammatory (Lee et al., 2011[8]), anti-microbial (Sleha et al.,[12]) and antiviral (Liu et al., 2019[9]) have reported XH to be a good candidate for AD and Huntington's disease. XH mediates its neuroprotective activity by inhibiting the inflammatory response, reducing the expression of TNFα, reducing iNOS expression, decreasing free radical formation, reducing overexpression of HIF-1α), activation, and apoptosis (i.e. active caspase-3, TNF-α, maintains the level of Bax/Bcl-2 ratio). It shows a considerable ROS scavenging property and hence is reported to have good antioxidant activity. Despite its prospects, XH has poor solubility and poor permeability which makes it less bioavailable (20 %). This makes its application limited. Being poorly permeable it cannot cross the blood-brain barrier and hence it cannot mediate its therapeutic activity. To overcome this challenge, novel formulations must be developed to uncover their unique and pivotal role in the management of PD. XH-loaded nanostructured lipid carriers (NLCs) can be formulated to boost bioavailability and have an efficient therapeutic effect against PD.

NLCs possess key characteristics for drug treatment such as increased solubility, improved permeability (intestinal permeability/blood-brain barrier permeability), site targeting potential, long-term drug storage stability, controlled particle size, and efficient drug entrapment. NLCs contain solid lipids, liquid lipids, and surfactants. They improve the bioavailability as their drug degradation in GIT is reduced to greater extents. They increase the permeability and reduce the first-pass metabolism. Additionally, they get converted from crystalline form to amorphous form when loaded into NLCs which accounts for increased solubility. Therefore, NLCs formulation of XH will deliver maximum amounts of the drug at the target site which in turn would enhance the therapeutic activity of XH in the treatment of PD.

On the other hand, short-chain volatile fatty acid, butyric acid (CH_3_CH_2_CH_2_COOH), is produced by anaerobic bacteria naturally and has significant uses in the chemical, culinary, pharmaceutical, and animal feed sectors. Butyric acid has a wide range of impacts on human energy homeostasis, inflammation, oxidative stress, immunological function, and related disorders (such as diabetes and obesity), as well as strong antibacterial and anticarcinogenic properties. Butyrate has been postulated as a neuroprotectant and has been shown to positively influence mitochondrial function, including boosting oxidative phosphorylation and beta-oxidation (Jiang et al., 2018[7]). Butyric acid plays a significant role in the local (in the gut) and systemic (through circulating butyrate) maintenance of immunological homeostasis. It has been noted that butyric acid inhibits specific cytokines that promote inflammation. Therefore, lack or deficiency of these in the gut may contribute to an overactive inflammatory response. The integrity of the intestinal epithelial barrier is likewise safeguarded by butyrate in the gut. Therefore, decreased butyric acid levels result in a compromised or unresponsive intestinal epithelial barrier. The ability of butyric acid to reduce inflammation and oxidative stress has been thoroughly examined and validated by numerous studies. However, the exact method by which microorganism-produced butyrate accelerates the development of regulatory T cells is unknown. Sodium butyrate NaB is reported to decrease the expression of α-synuclein in substantia nigra, protecting against rotenone-induced loss of dopaminergic neurons (Zhang et al., 2023[15]), and ameliorates motor and behavioral deficits (Cristiano et al., 2022[3]). 

The overall hypothesis relies on the facts related to the utilization of XH-loaded NLCs for the treatment of PD in combination with butyric acid. This can be followed by the addition of butyric acid in the developed NLCs which offer a homogeneous dispersion. To this dispersion cryoprotectants such as lactose/dextrose/maltose can be added. These sugar moieties will play the dual role of cryoprotectant as well as adsorbent for residual moisture post-lyophilization leading to free-flowing powder. Thus, upon reaching GIT, the XH will get absorbed via the intestine to the systemic circulation and will be carried out to the brain, where being a lipidic nanoparticle it will easily penetrate BBB and the XH will elicit its action. Simultaneously, butyric acid administered as a postbiotic will elicit its anti-PD, anti-oxidant, and anti-inflammatory action via the gut-brain axis. The diagrammatic representation of the mechanism of action of XH and butyric acid is represented in Supplementary Figure 1.

### Limitations of the hypothesis

While the hypothesis of using XH-loaded NLCs with butyric acid for PD treatment is promising, several limitations exist. The bioavailability and stability of both compounds can vary among individuals, impacting therapeutic outcomes. Additionally, the variability in natural product concentrations due to cultivation and extraction methods may lead to inconsistent effects and complicate dosing. While butyric acid has potential benefits for gut health and inflammation, its neuroprotective role is not fully understood, requiring further research. Thus, while this approach is innovative, it is crucial to evaluate these limitations and conduct rigorous testing to ensure reliable therapeutic results.

## Conflict of interest

The authors declare no conflict of interest.

## Supplementary Material

Supplementary information

## References

[1] Alves G, Müller B, Herlofson K, HogenEsch I, Telstad W, Aarsland D (2009). Incidence of Parkinson’s disease in Norway: The Norwegian ParkWest study. J Neurol Neurosurg Psychiatry.

[2] Brakedal B, Toker L, Haugarvoll K, Disease CTP (2022). A nationwide study of the incidence, prevalence and mortality of Parkinson’s disease in the Norwegian population. NPJ Park Dis.

[3] Cristiano C, Cuozzo M, Coretti L, Liguori FM, Cimmino F, Turco L (2022). Oral sodium butyrate supplementation ameliorates paclitaxel-induced behavioral and intestinal dysfunction. Biomed Pharmacother.

[4] Hartkorn A, Hoffmann F, Ajamieh H, Vogel S, Heilmann J, Gerbes AL (2009). Antioxidant effects of xanthohumol and functional impact on hepatic ischemia-reperfusion injury. J Nat Prod.

[5] Je G, Arora S, Raithatha S, Barrette R, Valizadeh N, Shah U (2021). Epidemiology of Parkinson’s Disease in Rural Gujarat, India. Neuroepidemiology.

[6] Jiang CH, Sun TL, Xiang DX, Wei SS, Li WQ (2018). Anticancer activity and mechanism of xanthohumol: A prenylated flavonoid from hops (Humulus lupulus L.). Front Pharmacol.

[7] Jiang L, Fu H, Yang HK, Xu W, Wang J, Yang S-T (2018). Butyric acid: applications and recent advances in its bioproduction. Biotechnol Adv.

[8] Lee IS, Lim J, Gal J, Kang JC, Kim HJ, Kang BY (2011). Anti-inflammatory activity of xanthohumol involves heme oxygenase-1 induction via NRF2-ARE signaling in microglial BV2 cells. Neurochem Int.

[9] Liu X, Bai J, Jiang C, Song Z, Zhao Y, Nauwynck H (2019). Therapeutic effect of Xanthohumol against highly pathogenic porcine reproductive and respiratory syndrome viruses. Vet Microbiol.

[10] Poewe W, Antonini A, Zijlmans JC, Burkhard PR, Vingerhoets F (2010). Levodopa in the treatment of Parkinson’s disease: an old drug still going strong. Clin Interv Aging.

[11] Rus T, Premzl M, Križnar NZ, Kramberger MG, Rajnar R, Ocepek L (2022). Adverse effects of levodopa/ carbidopa intrajejunal gel treatment: A single-center long-term follow-up study. Acta Neurol Scand.

[12] Sleha R, Radochova V, Mikyska A, Houska M, Bolehovska R, Janovska S (2021). Strong antimicrobial effects of xanthohumol and beta-acids from hops against Clostridioides difficile infection in vivo. Antibiotics (Basel).

[13] Wang X, Ho SL, Poon CY, Yan T, Li HW, Wong MS (2019). Amyloid-β Aggregation inhibitory and neuroprotective effects of xanthohumol and its derivatives for Alzheimer's diseases. Curr Alzheimer Res.

[14] Wei Z, Li X, Li X, Liu Q, Cheng Y (2018). Oxidative stress in Parkinson’s disease: a systematic review and meta-analysis. Front Mol Neurosci.

[15] Zhang Y, Xu S, Qian Y, Mo C, Ai P, Yang X (2023). Sodium butyrate ameliorates gut dysfunction and motor deficits in a mouse model of Parkinson’s disease by regulating gut microbiota. Front Aging Neurosci.

