# Supplementary material for: Combination therapy of phytonanomedicine and post-biotic for the management of Parkinson’s disease
Source: EXCLI J. 2024 Oct 10;23:1234–7. doi: 10.17179/excli2024-7770 (PMC11609944; doi:10.17179/excli2024-7770)
Supplement: Supplementary information [file EXCLI-23-1234-s-001.pdf]

EXCLI Journal 2024;23:1234-1237 – ISSN 1611-2156
Received: August 24, 2024, accepted: September 26, 2024, published: October 10, 2024

Supplementary information to:

Letter to the editor:

COMBINATION THERAPY OF PHYTONANOMEDICINE AND POST-BIOTIC FOR THE MANAGEMENT OF PARKINSON'S DISEASE

Bushra Bashir[a], Sukriti Vishwas[a], Monica Gulati[a,b], Gaurav Gupta[c,d], Sachin Kumar Singh[a,b,*]

[a] School of Pharmaceutical Sciences, Lovely Professional University, Phagwara, Punjab, India
[b] Faculty of Health, Australian Research Center in Complementary and Integrative Medicine, University of Technology Sydney, Ultimo, NSW 2007, Australia
[c] Center of Medical and Bio-allied Health Sciences Research, Ajman University, Ajman, United Arab Emirates
[d] Center for Research Impact & Outcome-Chitkara College of Pharmacy, Chitkara University, Punjab, India

* **Corresponding author:** Sachin Kumar Singh, School of Pharmaceutical Sciences, Lovely Professional University, Phagwara-144411, Punjab, India. Tel.: +91-9888720835, E-mail: singhsachin23@gmail.com

https://dx.doi.org/10.17179/excli2024-7770

This is an Open Access article distributed under the terms of the Creative Commons Attribution License (http://creativecommons.org/licenses/by/4.0/).

EXCLI Journal 2024;23:1234-1237 – ISSN 1611-2156
Received: August 24, 2024, accepted: September 26, 2024, published: October 10, 2024

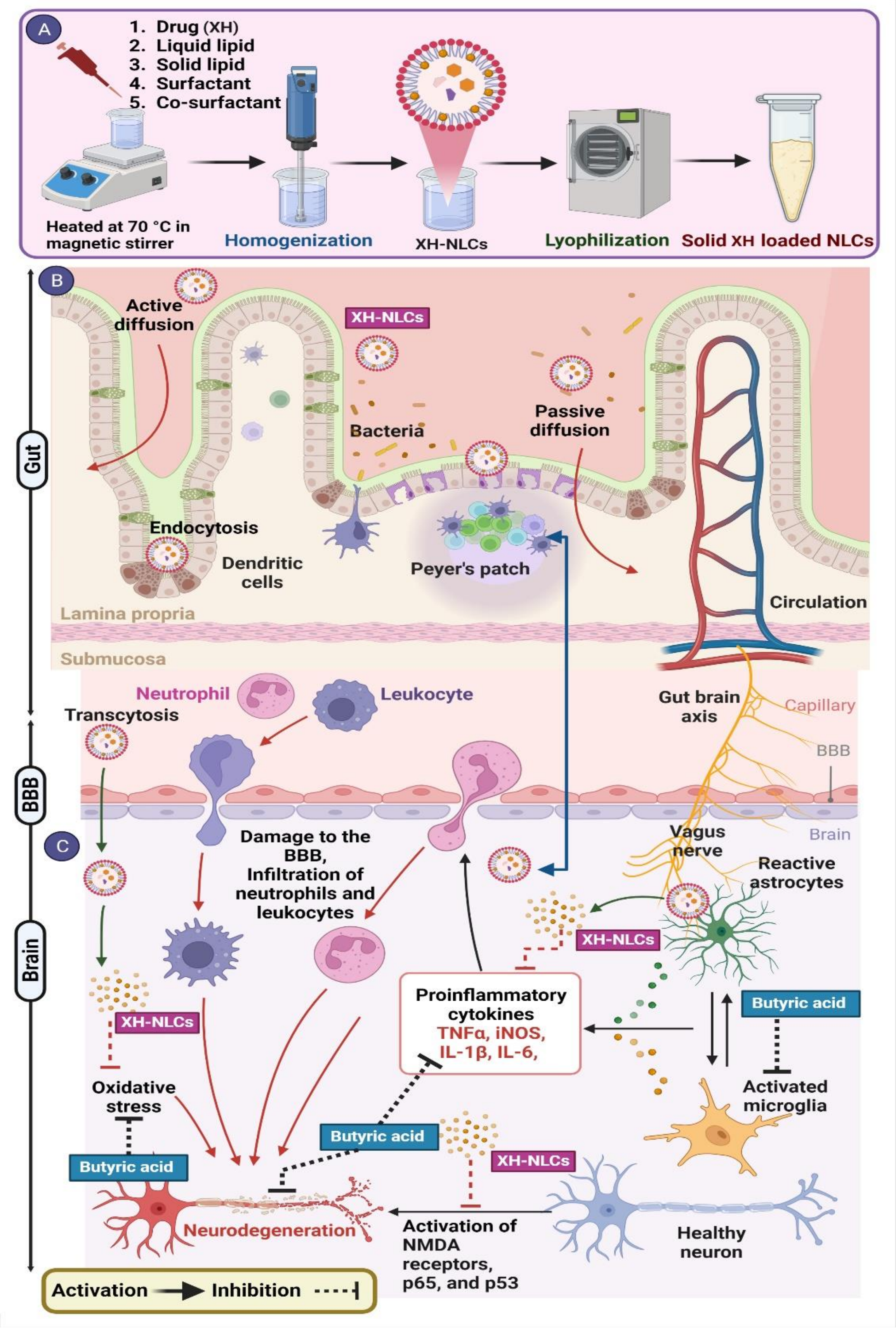

Supplementary Figure 1: Figure demonstrating **(A)** Formulation of XH-NLCs, **(B)** Absorption of NLCs through gut membrane, **(C)** Mechanism involved in delivery of XH-NLCs and butyric acid
